# The effect of doping, anti-doping measures, and sociological factors on the annual distribution of all-time top performances in short, middle, and long-distance running

**DOI:** 10.3389/fspor.2025.1683718

**Published:** 2025-11-21

**Authors:** Dora Dragcevic, Jelena Jaksic, Lukas Libric, Ozana Jaksic, Delfa Radic-Kristo, Vlatka Pandzic, Ozren Jaksic

**Affiliations:** 1 Department of Hematology, University Hospital Merkur, Zagreb, Croatia; 2 School of Medicine, University of Zagreb, Zagreb, Croatia; 3 School of Medicine, University of Josip Juraj Strossmayer Osijek, Osijek, Croatia; 4 Department of Endocrinology, Diabetes, Metabolic Diseases and Clinical Pharmacology, University Hospital Dubrava, Zagreb, Croatia; 5 Department of Hematology, University Hospital Dubrava, Zagreb, Croatia

**Keywords:** androgen anabolic steroids, Athlete Biological Passport, blood doping, erythropoietin, running, testosterone, tetrahydrogestrinone, top performance

## Abstract

Scientific advancements, doping, anti-doping strategies, and sociological factors all influence athletic performance. This retrospective study aimed to analyze how different variables affect the annual distribution of all-time top performances from 1968–2023 in men's and women's running disciplines with different physiological demands (100 m–anaerobic, 5,000 m–aerobic, 800 m–mixed aerobic-anaerobic). A public database, https://www.alltime-athletics.com, with results linked to doping already excluded, was used. The annual distribution of top performances was modeled using negative binomial distribution to assess the influence of continuous and categorical variables, including doping availability (e.g., tetrahydrogestrinone, erythropoietin), anti-doping measures (e.g., Athlete Biological Passport modules, out-of-competition testing), and sociological factors (e.g., Olympic years). Steroid doping related variables showed significant effects on the annual distribution of top performances across almost all tested men's and women's disciplines, with a significant negative influence of testosterone limitations in women's 800 m, as well as a negative influence of the steroid module of the Athlete Biological Passport in women's 100 m and men's 800 m and 5,000 m disciplines. Blood doping related variables, including the positive influence of erythropoietin availability and the negative influence of the hematological module of the Athlete Biological Passport, demonstrated significant effects on performance in both 800 m disciplines. Olympic years emerged as a positive factor in the majority of tested disciplines, while the COVID-19 pandemic showed no significant influence. Despite strict anti-doping measures, our findings reveal a complex interaction of doping practices, regulatory frameworks, and social factors that continue to influence trends in elite athletic performance.

## 1 Introduction

**Figure 1:**
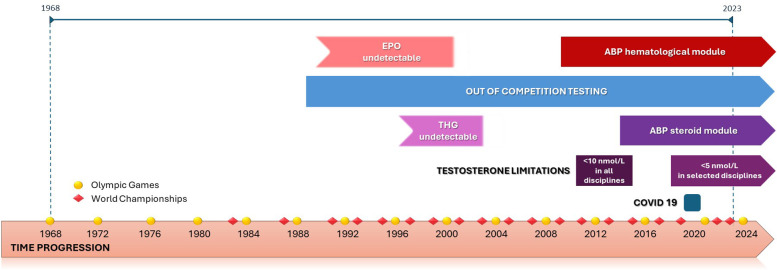
The schematic timeline of the tested factors influencing athlete's top performance. Legend: time progression, years of competition (1968–2023); out-of-competition testing, period with out-of-competition testing (approved in 1989 by International Olympic Committee, implemented in major competitions since 1990); Epo undetectable, unavailable erythropoietin detection (1990s—2000); THG undetectable, unavailable tetrahydrogestrinone detection (1990s—mid-2003); ABP—hematological module, years with hematological module of the Athlete Biological Passport (from 2009); ABP—steroid module, years with steroid module of the Athlete Biological Passport (from 2014); testosterone limitation < 10 nmol/L, testosterone limitation of 10 nmol/L in all women's disciplines (2011–2015); testosterone limitation < 5 nmol/L, testosterone limitation of 5 nmol/L in selected women's disciplines (from 2019); COVID-19 pandemic, lockdown year due to COVID-19 (2020); World Championships, years with World Athletics Championships (every 2 years, except for the first three competitions that were held every 4 years and 2021 World Championship held in 2022); Olympic Games, years with Olympic Games (every 4 years, except for 2020 Olympic games held in 2021).

The intrinsic value of sport lies in striving for excellence while respecting the athlete's health, promoting genuine and equal competition based on natural talent, and enacting fair rules (1). Improving athletic performance and results is closely linked to advances in the knowledge of human physiology and sports science, leading to more effective training and better results. Some improvement in sports performance may be related to doping abuse, whether through substances or methods (1). The history of doping and anti-doping has been a constant and long-standing race to conceal and detect these unfair influences, with the debut of concrete anti-doping testing in the 1980s (2). One example of the earliest and most significant measures introduced to improve control over prohibited substances and methods was the introduction of out-of-competition (OOC) testing from the 1990s (2). Another turning point in the aspect of blood doping was the ban of erythropoietin (Epo) utilization in 1990, followed by the implementation of a direct detection method for its abuse in 2000. Nevertheless, some data in endurance sports indicated a continuous use of Epo and its analogues in advanced dosing schedules, afterwards (3, 4). As these direct detection methods were insufficient to uncover all aspects of blood doping (e.g., Epo microdose, autologous blood transfusion), the Athlete Biological Passport (ABP), which monitors biological parameters that can be influenced by external factors, was first introduced in 2009 with its hematological module (5–8). The positive experience with the hematological module was followed by the introduction of the steroid module of the ABP in 2014, aimed at improving the indirect detection of androgen anabolic steroids (AAS) (2, 8, 9). A notable example of a designer substance used in a doping conspiracy among the most elite athletes was the synthetic AAS tetrahydrogestrinone (THG), commonly known as “The Clear” (10). It could not be detected until mid-2003 when a small amount of the substance was provided to the United States Anti-Doping Agency as part of the BALCO operation (10, 11). The exact year when THG entered elite sport is unclear, but several athletes who achieved their top performances in the late 1990s (e.g., in the men's 100 m category in 1996) were later officially related to its utilization (12). AAS have proven effective in both men's and women's sports, with particular emphasis on the competitive advantage of athletes with naturally occurring very high testosterone levels competing in women's categories (13–15). The highest-profile example worldwide in women's athletics involved the Olympic gold medalist in middle-distance running, which led to the first implementation of testosterone limitation by the International Association of Athletics Federations (IAAF) from 2011–2015 (16). During this period, all participants in the women's categories were required to have a testosterone concentration below 10 nmol/L (17). After the Court of Arbitration for Sport (CAS) suspended this decision, a period of unrestricted competition, i.e., without testosterone limitation, in women's categories followed (2, 18). A new turning point in women's sport occurred in 2018 with the IAAF's adoption of modified limitations addressing eligibility to compete in the women's category for athletes with differences of sex development (DSD), requiring testosterone levels to be below 5 nmol/L for at least 6 months before competing in selected women's categories. Although it was planned to adopt these regulations in November 2018, due to many challenges, the regulations were implemented in May 2019 (18, 19). The challenges of detecting doping, using either direct or indirect methods, all while ensuring fair competition, are also highlighted in research that investigates performance profiling as one of potential detection approaches (20–22). Meanwhile, external factors may also influence the number of top performances in both men's and women's sports. During the COVID-19 pandemic lockdown, the effectiveness of the anti-doping measures may have been significantly reduced due to the disruption of in-competition (IC) testing because of canceled competitions, and OOC testing. Canceled competitions and training could also have affected sports performance (23). All of this highlights the challenges faced in detecting and preventing doping in sports. The chronological sequence of specific doping abuse and turning points in anti-doping efficacy might directly affect the number of top performances in one particular year. Analysis of athletic performance has shown that better results are achieved on or around the major competitions [i.e., Olympic Games or World Championship (WC)] in multiple sports disciplines (24), which may be the result of both targeting peak performance and/or doping utilization, whose true prevalence in elite sport is hard to establish and ranges from below 5% up to 30% in various studies (25–29). The simple progression of time, as a variable encompassing a wide range of factors and improvements that can develop over the years, can also influence athletic performance (30, 31). In exploring the dynamics of elite athletic achievement, the primary objective of our study was to identify potential factors associated with the temporal distribution of top performances in various men's and women's running categories (Figure 1).

## 2 Methods

**Figure 2:**
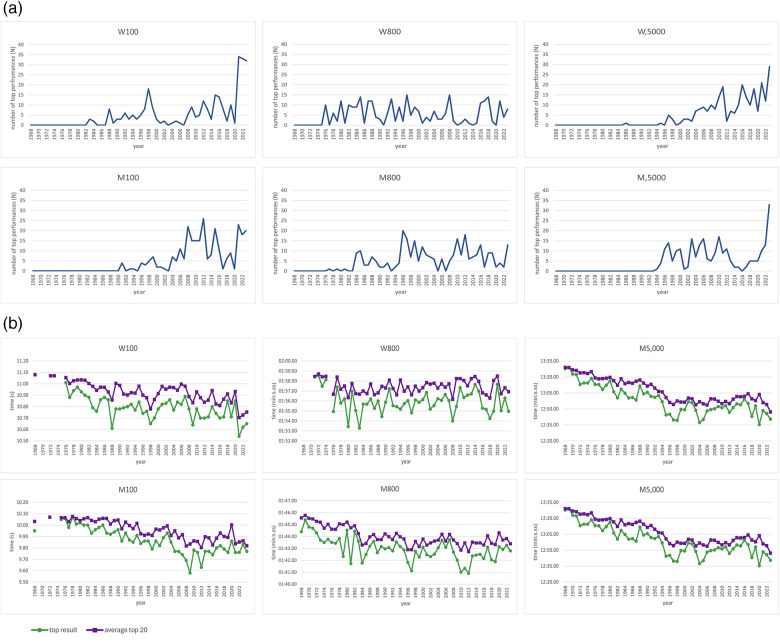
The annual distribution of all-time top performances in women's and men's running disciplines and trends in performance during the tested period. Legend: **(a)** The annual distribution of all-time top performances in tested men's and women's disciplines. **(b)** The trends of performance in tested discipline (best results per year and best 20 results per year). W100 m, women's 100 m discipline; W800 m, women's 800 m discipline; W5,000, women's 5,000 m discipline, M100m, men's 100 m discipline; M800 m, men's 800 m discipline; M5,000 m, men's 5,000 m discipline; number of top performances (N), the annual number of all-time top performances meeting the inclusion criteria; time (s), time of the performance in each year in seconds top result, time of the top result per year; average top 20, average time of top 20 performances per year (in the years with less than 20 performances calculations were made with all results); time (min:s.ss), time of the performance in format minutes:seconds.centiseconds.

The annual distribution of the world's top performances meeting inclusion criteria was analyzed (Supplementary Table S1) (32). The period covered ranged from 01/01/1968 to 31/12/2023. Both men's and women's outdoor running disciplines, with distinct physiological requirements, were included in the study as representative examples: short-distance (100 m), middle-distance (800 m), and long-distance (5,000 m) (33, 34). The study included results from all disciplines ranked at or below 250th position at the table of all-time top performances, where the time of the last included result was set as a chosen time limit for each discipline. The total number of all-time top performances had to be determined separately for each discipline, considering the substantial number of results at the same or very close times. All results meeting the criteria were included, regardless of whether they were from the same athlete or not (Supplementary Table S1). Finally, the subsets of approximately 250–300 top results in each discipline were identified to minimize the difference between the analyzed groups (Figure 2). The publicly available web database (https://www.alltime-athletics.com) of all-time best top performances in outdoor running served as the data source (35). Results previously linked to doping use are excluded from this database (Supplementary Figure S1). The effects of the following variables were tested: time progression—years of competition (as a continuous variable, from 1968–2023) and further categorical variables: years with implementation of the ABP—hematological module: 2009–2023; years with implementation of the ABP—steroid module: 2014–2023; years without Epo detection (Epo—undetectable): 1990–2000; years without THG detection (THG—undetectable): 1996–mid-2003; years with spread OOC testing: 1990–2023; years of Olympic Games (Olympics); years of WC; COVID-19 pandemic: year 2020. For women, two periods of testosterone limitation were applied according to the implemented testosterone rules in different disciplines, i.e., for 100 m and 5,000 m: 2011–2015 (testosterone limitation—100 and 5,000 m) and for 800 m (testosterone limitation—800 m): 2011–2015 and 2019–2023. Spearman correlation was performed to compare the annual number of all-time top performances between different disciplines (100 m, 800 m, 5,000 m) for men and women separately, as well as to compare the annual number of all-time top performances between men and women for the same discipline. Each analysis was conducted for the entire period (1968–2023) and the period between the year of the first top performance of the analyzed disciplines' pair and 2023 (36). The selected pairs represented combinations of sprint, middle-distance, and long-distance events, including both intra- and inter-gender comparisons. The second analysis was performed to diminish the potential positive effect of years without top performances (count 0) on the correlation coefficient. A modification was done for the women's 5,000 m discipline, where the period from the year of the first top result (1981–2023) and the period from the start of the official competitions (1995–2023) were analyzed. The reason was that before 1995, the long-distance women's discipline in all major championship events (i.e., Olympics, WC) was a 3,000 m race (35, 37). The negative binomial distribution was used to test the potential influence of multiple different continuous and categorical variables (time progression, ABP—hematological module, ABP—steroid module, Epo—undetectable, testosterone limitation—800 m, testosterone limitation—100 m and 5,000 m, THG—undetectable, OOC testing, WC, Olympics, COVID-19 pandemic) on the number of top performances in the men's and women's disciplines. Calculated alpha (*α*) and its *p*-value confirmed its superiority to Poisson regression on our dataset (Supplementary Table S2). As the analyses included count data, the pseudo-R-square was used to evaluate goodness of fit and to interpret correlations. Pseudo R-square values between 0.2 and 0.4 represent a reasonable fit, i.e., the closer the values are to 1, the better the fit of the model is (38–40). Incidence rate ratios (IRR) and their 95% confidence intervals (CI) were reported. All analyses were performed using Stata 17 statistical software.

## 3 Results

The annual distribution of all-time top performances meeting the inclusion criteria per discipline is shown in Figure 2. The Spearman correlation for the annual distribution of all-time top performances meeting the inclusion criteria in the different women's disciplines showed a significant positive correlation between the number of top performances in the 100 m and the number of top performances in the 5,000 m discipline for both periods analyzed and the first analyzed period in the 100 m and 800 m disciplines. The same analyses showed no significant correlation between the annual distribution of all-time top performances in the women's 800 m discipline and the annual number of all-time top performances in the 5,000 m discipline and second period in the 100 m discipline (Table 1A). For men, a significant correlation was confirmed between the annual number of all-time top performances in all disciplines and time periods (Table 1B). The analysis of the annual distribution of all-time top performances in the same discipline but in a different category (men vs. women) showed a significant correlation in both tested periods in the 100 m discipline and for the first tested period in 5,000 m discipline (1981–2023) (Table 1C). In contrast, there was no significant correlation in the second examined period for the 5,000 m disciplines (1995–2023), as well as in both examined periods for the 800 m disciplines. Multivariate analysis of factors influencing the annual distribution of all-time top performances in various disciplines for men and women showed different sets of associated specific variables within each discipline (Table 2). In the men's 100 m and 5,000 m running disciplines the likelihood surface was unstable (non-concavity warnings), so results are reported but should be interpreted with caution. In the women's 100 m discipline, the annual distribution of all-time top performances was positively associated with time progression, undetectable Epo, testosterone limitations—100 m and 800 m and Olympics, while negative association was confirmed for ABP—steroid module and OOC. In the men's 100 m a positive association was found for time progression and Olympics. In the women's 800 m discipline, a positive influence was found for time progression, undetectable Epo, WC and Olympics with a negative influence for ABP—hematological module, testosterone limitation—800 m and OOC. In the men's 800 m discipline, a positive influence was found for the time progression, undetectable THG, undetectable Epo, and Olympics with a negative influence for ABP—steroid module and OOC testing. In the women's 5,000 m discipline, only time progression showed a significant influence which was positive. This is probably a result of these disciplines historical development explained previously. Finally, in the men's 5,000 m discipline, time progression and undetectable THG were identified as positive influences, whereas ABP—steroid module was a negative factor.

**Table 1:** A–C paired correlations between the annual distribution of all-time top performances in running disciplines.

Table 1A Spearman's correlations between the annual distribution of all-time top performances of different running disciplines in women			
	W100 m	W800 m	W5,000 m
W100 m	1		
W800 m	0.30, *p* = 0.025, (56), (1968–2023)	1	
	0.06, n.s., (48), (1976–2023)*		
W5,000 m	0.44, *p* = 0.004, (43), (1981–2023)**	−0.08, n.s., (43), (1981–2023)**	1
	0.38, *p* = 0.040, (29), (1995 -2023)***	0.09, n.s., (29), (1995–2023)***	
**Table 1B Spearman's correlations between the annual distribution of all-time top performances of different running disciplines in men**			
	M100 m	M800 m	M5,000 m
M100 m	1		
M800 m	0.58, *p* < 0.001, (56), (1968–2023)	1	
	0.47, *p* < 0.001, (47), (1977–2023)*		
M5,000 m	0.77, *p* < 0.001, (56), (1968–2023)	0.57, *p* < 0.001, (56), (1968–2023)	1
	0.37, *p* = 0.033, (33), (1991–2023)*	0.44, *p* = 0.002, (47), (1977–2023)*	
**Table 1C Spearman's correlations between the annual distribution of all-time top performances in the same women's and men's running disciplines**			
	M100 m	M800 m	M5,000 m
W100 m	0.74, *p* < 0.001, (56), (1968–2023)		
	0.58, *p* < 0.001, (41), (1983–2023)*		
W800 m		0.24, n.s., (56), (1968–2023)	
		−0.04, n.s., (48), (1976–2023)*	
W5,000 m			0.58, p˂0.001, (43), (1981–2023)**
			0.00, n.s., (29), (1995–2023)***

Legend: The results are expressed as: rho, Spearman's rho correlation coefficient; *n*. s., not significant (*p* > 0.05) or *p* values; number of top performances in the tested period (*n*), period (yyyy—yyyy). The first row in each discipline presents the results for the whole tested period, whereas the second row presents the results for the period from the first top result.

W100 m, women's 100 m discipline; W800 m, women's 800 m discipline; W5,000, women's 5,000 m discipline; M100 m, men's 100 m discipline; M800 m, men's 800 m discipline; M5,000 m, men's 5,000 m discipline.

* Period from the first top result in either of the two compared categories to 2023 in all disciplines except women's 5,000 m.

** Period from the first top result in women's 5,000 m discipline.

*** Period from the debut in Olympic Games and World Championship competitions.

**Table 2:** Multivariate analysis of factors influencing the annual distribution of all-time top performances in men's and women's running disciplines.

Category Parameter	W100	W800	W5,000*^Δ^*	M100	M800	M5,000
	*pseudo* *R2 =* *0.30*	*pseudo R2* *=* *0.18*	*pseudo R2* *=* *0.35*	*pseudo R2* *=* *0.30*	*pseudo R2* *=* *0.30*	*pseudo R2* *=* *0.29*
Time progression	1.25 (1.15–1.35)*	1.18 (1.12–1.25)*	1.13 (1.02–1.25)	1.09 (1.00–1.18)	1.13 (1.06–1.20)*	1.16 (1.06–1.26)*
ABP—steroid module	0.19 (0.05–0.64)	2.97 (0.52–16.87)	0.44 (0.13–1.46)	0.36 (0.11–1.16)	0.25 (0.08–0.75)	0.16 (0.04–0.57)
ABP—hematological module	2.48 (0.83–7.47)	0.12 (0.02–0.61)	1.05 (0.42–2.62)	2.20 (0.69–6.97)	2.53 (0.75–8.51)	1.08 (0.32–3.67)
EPO—undetectable	46.42 (13.33–161.66)*	7.77 (3.21–18.81)*	0.45 (0.13–1.52)	1.01 (0.30–3.37)	2.74 (1.10–6.84)	1.21 (0.37–3.95)
THG—undetectable	1.02 (0.50–2.07)	0.76 (0.40–1.46)	0.99 (0.44–2.25)	0.94 (0.39–2.23)	3.97 (1.87–8.42)*	2.52 (1.03–6.18)
Testosterone limitation—100 m and 5,000 m	2.51 (1.01–6.24)	n/a	1.25 (0.48–3.27)	n/a	n/a	n/a
Testosterone limitation—800 m	n/a	0.19 (0.08–0.44)*	n/a	n/a	n/a	n/a
OOC testing	0.02 (0.00–0.10)*	0.02 (0.01–0.09)*	7.68 (0.39–152.13)	2.68 × 10⁷ (0–∞)!	0.12 (0.03–0.55)	8.64 × 10⁶ (0–∞)!
Olympics	2.80 (1.60–4.91)*	2.64 (1.67–4.18)*	0.99 (0.53–1.85)	2.25 (1.07–4.74)	1.89 (1.06–3.38)	1.51 (0.67–3.36)
WC	1.55 (0.94–2.56)	1.97 (1.29–3.01)	0.92 (0.54–1.58)	1.39 (0.72–2.69)	1.10 (0.65–1.87)	1.60 (0.81–3.14)
COVID-19 pandemic	0.84 (0.29–2.43)	0.57 (0.10–3.43)	1.15 (0.42–3.18)	0.62 (0.14–2.73)	0.94 (0.21–4.14)	0.46 (0.09–2.35)

Negative binomial distribution model with overdispersion parameter *α* = 0.13–0.42, all model *p* value ˂ 0.05 (detailed model related parameters are presented in Supplementary Table S2). Results are presented as incidence rate ratio (IRR) and 95% confidence intervals; significant results (*p* < 0.05) are highlighted in grey.

W100 m, women's 100 m discipline; W800 m, women's 800 m discipline; W5,000, women's 5,000 m discipline; M100 m, men's 100 m discipline; M800 m, men's 800 m discipline; M5,000 m, men's 5,000 m discipline.

Time progression: 1968–2023; ABP—hematological module, years with implementation of the hematological module of the Athlete Biological Passport: 2009–2023; ABP—steroid module, years with implementation of the Athlete Biological Passport: 2014–2023; Epo—undetectable, years without erythropoietin detection: 1990–2000; THG—undetectable, years without tetrahydrogestrinone detection: 1996–2002; OOC testing, years with out-of-competition testing: 1989–2023; Olympics, years of Olympic Games; WC, years of World Championships; COVID-19 pandemic, year 2020; testosterone limitation—100 and 5000 m, testosterone limitation in 100 m and 5,000 m discipline: 2011–2015 (excluding 800 m discipline); testosterone limitation—800 m, testosterone limitation in 800 m discipline: 2011–2015 and 2019–2023.

*Δ*—results from the year of inclusion in major official competitions to the end of data collection (1995–2023).

* *p* value ˂ 0.001.

!—unstable estimate.

pseudo R2—pseudo R-square (McFadden) for each tested category.

n/a—parameter not applicable in specific category.

## 4 Discussion

The top performances observed in elite sport consistently attract general and scientific interest, raising questions regarding the physiological, genetic, and potentially pharmacological factors behind these results. In examining the potential influence of various factors—including the availability of doping and anti-doping methods and regulations—our study analyzed the annual distribution of top performances in men's and women's running disciplines that have different physiological requirements. To meet those physiological demands, tailored doping measures might also be used. A short-distance running discipline (100 m) is a classic example of anaerobic activity, in which the creatinine phosphate and glucose-lactate system in white muscle fibers (type II, anaerobic) are crucial for energy production, and its innervation by a high number of neuromuscular units enables rapid activation in a short time (34, 41, 42). Short-distance performance is described by technique, maximal horizontal power output, and sprint-specific endurance, of which the last two are highly dependent on muscle properties that can be influenced by AAS (33, 41–43). AAS doping is mostly detected by direct detection methods, but novel and unknown substances can be detected with indirect methods, particularly the steroid module of the ABP. The physiologically opposite example is a long-distance running discipline (5,000 m), a classic example of aerobic activity in which sports performance is highly dependent on the multifactorial variable describing maximal oxygen uptake volume (VO2max). VO2max is a variable that comprises environmental conditions, lung capacity, oxygen delivery to the working muscle (blood hemoglobin concentration and maximal cardiac output), and skeletal muscle characteristics (mitochondrial density and the muscle ability to extract oxygen) (5, 34, 41). Of all the parameters mentioned, hemoglobin concentration is the easiest to influence. It has been shown to have a positive correlation with aerobic performance and can be influenced by various aspects of doping, including Epo, autologous blood transfusions, and hypoxia inducible factors (HIF) whose abuse is tested using both direct and indirect detection methods (e.g., hematological module of the ABP) (5, 44, 45). The middle-distance running discipline (800 m) lies somewhere in between and relies on both aerobic and anaerobic capacities because the energy produced in the anaerobic pathways is sufficient to ensure maximal power output for 10–15 s so in a run which lasts between 100 and 150 s, a certain proportion of the energy must be generated in the aerobic pathway (34, 46). It is therefore an example of a mixed aerobic and anaerobic activity in which both steroid and blood doping, as well as their detection methods, may have a significant influence (5, 34, 41).

In this context, our analysis highlighted the reflection of several distinct types of doping and anti-doping measures on the annual number of all-time top performances in specific disciplines. The possible effect of steroid doping was recognized, especially in women's anaerobic running disciplines. In women's 100 m discipline, the implementation of the steroid module of the ABP in 2014 showed a significant negative effect on the number of top performances, as well as OOC testing. Although there is no study directly addressing the specific anabolic effects of AAS in women, existing evidence suggests that, due to lower baseline androgen levels, women may exhibit relatively greater performance gains from AAS compared to men (47, 48). A study focusing primarily on anaerobic athletic disciplines showed a significant negative influence of improved AAS detection methods on the performance of athletes from countries known for state-sponsored doping who are competing in women's categories (49). Another study on the anaerobic sport (weightlifting) showed a significant decrease in athletes' performances after long-term detection of AAS doping (2016–2022) when compared to the prior period 2009–2015 (50). Taking above mentioned in consideration together with the results of the study comparing performance in women's categories with testosterone levels in which higher values showed no competitive advantage (51), significant positive influence of testosterone limitations below 10 nmol/L in women's 100 m category may be the results of confounding strategical use of AAS prior to the implementation of steroid module of the ABP or some other competition/technological influence. The exact explanation of this finding is complex and requires further investigation. The influence of steroid doping seems to be recognized also in the men's and women's 800 m discipline, which is a combination of aerobic and anaerobic activity. The undetectable THG showed a significant positive effect in men's 800 m discipline, whereas testosterone limitations showed significant negative influence in women's 800 m discipline. Although aerobic events such as the 5,000 m run are generally considered more susceptible to the effects of blood doping, AAS use also exerts broader physiological effects—most notably by stimulating erythropoiesis—which can enhance aerobic performance (52, 53). This mechanism may help explain our finding that the introduction of the steroid module of the ABP had a significant negative effect and undetectable THG significant positive effect on performance trends in the men's 5,000 m discipline, simultaneously with the lack of the significant influence of blood doping related variables. It is interesting to note that in women's 800 m discipline, the implementation of the steroid module of the ABP in 2014 did not show any statistical significance. This could be a bias of the discussions related to the significant negative effect of the implementation of testosterone limitations in preceding years, as well as the considerations about women's category eligibility, during the same period, which may have led athletes competing in the women's category to avoid testosterone/AAS doping. The controversies surrounding the IAAF testosterone rule suspension in 2015 and the results of the 2016 summer Olympics without any limitations led to a study that aimed to correlate measured testosterone levels in athletes competing in women's categories with their performance. This study included 1,332 blood samples from women's categories and 795 blood samples from men's categories collected during the 2011 and 2013 WC in various athletic disciplines (50). The data analysis revealed a significant positive correlation in athletes competing in women's categories with high testosterone levels in specific disciplines (i.e., 400 m, 400 m hurdles, 800 m, hammer throw, and pole vault), ranging from 1.8%–4.5% (50, 54). One of the disciplines where better results showed a significant positive correlation with detectable testosterone levels was the women's 800 m discipline (53). As a result, the IAAF adopted a rule mandating a testosterone threshold of 5 nmol/L for all competitors in women's running disciplines from 400 m–1 mile (18). In our study, the effect of a testosterone limitation at 10 nmol/L from 2011–2015 and 5 nmol/L from 2018 on the annual number of all-time top performances in the women's 800 m discipline was tested, and the significant negative effect of the testosterone limit in this discipline was confirmed—similar to the IAAF study (2, 54).

Our analysis also suggests that blood doping primarily aimed at enhancing aerobic capacity may have contributed to top athletic performances, with its impact shaped by the timing and effectiveness of detection methods. In the 800 m races, where the aerobic component is also essential, results showed the positive influence of doping with Epo and its analogs in both the men's and women's disciplines. This effect was more pronounced in the women's discipline, where the significant negative effect of the hematological module of the ABP was also observed. In the women's 5,000 m discipline, statistically significant influence was not confirmed for any of the tested variables, apart from positive effect of time progression. As 5,000 m race is an official long-distance discipline in major championship events since 1995 and when analyzing the annual distribution of all-time women's top performances in the 5,000 m discipline, there are few top results before the debut of any tested variables, which may explain the lack of a statistically significant influence (37). One interesting finding in the women's 100 m discipline is a significant influence of the period with undetectable Epo on the distribution of top performances. This could be explained by a cluster of top athletes performing in the time, overlapping with the period of the widespread utilization of different doping substances or could be related to improved recovery process, but warrants further investigation and is beyond the scope of this article (Supplementary Figure S1) (55–57).

Furthermore, the organization of anti-doping testing, major championship events, and some sociological and technological factors may influence athletes and their performance. Time progression covering wide range of technological, training and competition changes (e.g., super shoe era, track changes, pacing tactics, altitude training) showed a significant positive influence in all tested categories, similarly to several other studies which have confirmed positive effects of technological advancement on athletes' performance (5, 58, 59). At the beginning of the anti-doping fight, all testing was done IC, which allowed athletes and their teams to carefully plan and calculate the use of prohibited substances and methods OOC, to achieve an edge in sports performance without being caught (2). After the adoption of Anti-Doping Convention in 1989, the implementation of OOC testing by major international federations from 1990 has had a significant negative effect on the men's and women's 800 m running discipline and women's 100 m running discipline (60). This result can be compared to the findings of Collantes et al., who reported a sharp decline in performance, particularly in women's events like the 800 m, after 1990 (31). They primarily attributed this trend to the presumed end of state-sponsored doping programs, for instance in the German Democratic Republic, which was known for its systematic use of AAS with prominent results in women (31). While we acknowledge the significance of the political shift, we emphasize that its primary impact lay in the introduction of the exceptionally effective anti-doping measure, as OOC testing is, which effectively curbed the widespread global use of AAS (61, 62). The use of more refined and targeted variables in our study, such as undetectable THG, showed that the use and effect of AAS persisted even after 1990, when new designer substances escaped OOC testing.

In the Olympic years, the chance of achieving a top result in both the men's and women's 100 m and 800 m run is greater than in the non-Olympic years. Whether this is solely due to better preparation and focus on this one competition, or whether there is also undetected use of doping substances, and how the anti-doping fight affects this result, cannot be assumed (63, 64). Several studies examining potential factors that influence Olympic performance have concluded that population, per capita income, hosting the Olympic Games, and political ideology may influence the results (65, 66). Although some recent studies using new statistical approaches have concluded that only hosting the Olympic Games and the country's population remain essential factors, the success is multifactorial (67, 68). Finally, it should be noted that the COVID-19 pandemic, a complex variable that may incorporate many other factors (e.g., suspended competitions, special health measures, irregular training, suspended anti-doping testing, etc.) did not significantly affect the number of top performances across tested men's and women's disciplines (69, 70).

In the end, looking at the annual number of top performances between the different categories in both sexes, the women's 800 m running category shows no significant correlation with other categories. In comparison, the men's 800 m running category shows a significant correlation with the tested categories and similar signals as the 100 m and 5,000 m running categories. The explanation for this could lie in the significant effect of the eligibility criteria for women in this discipline, but the exact reason remains to be determined. On the other hand, the other two disciplines, which are on opposite sides of the physiological requirements, have a significant correlation with each other, showing clear doping cycles with a higher number of top performances in years when doping substances are undetectable and a significant decrease after the introduction of novel detection methods. There are certainly many other factors that could be confounding factors (e.g., super shoe era, track changes, pacing tactic), so these results require further scientific analysis.

This study has several limitations. Due to its retrospective and observational design, causal relationships cannot be established. The complex nature of sports performance, influenced by overlapping and often unrecognized physiological, technological, and sociological factors, limits the ability to isolate individual effects and may conceal potential confounders. We relied on publicly available data, which may include unverified performances. Proxy variables, such as the undetectable THG or the introduction of ABP, were used to estimate doping-related influences, however, they did not directly measure actual usage or enforcement. Although known doping cases were excluded, the number of undetected cases remain unknown. Therefore, our findings indicate associations, and we have interpreted them with appropriate caution. To address these limitations, we developed refined and targeted proxy variables, which ultimately revealed meaningful associations despite the constraints of the available data.

## 5 Conclusion

This study evaluated how doping, its detection methods, and regulations affect the annual distribution of top athletic performances. The findings suggest that both doping practices and the effectiveness of anti-doping measures have significantly shaped elite performance trends over the past five decades, even after confirmed doping cases were excluded. Using refined proxy variables, we identified measurable associations between the availability of substances like THG and Epo and increased prevalence of top performances, particularly in events aligned with their physiological influence. Anti-doping interventions, including the ABP modules and OOC testing, appeared to mitigate these effects. Testosterone regulations in women's middle—distance running events were also linked to diminished performance levels. Sociological factors, such as the Olympic Games and WC, further influenced performance trends, underscoring the multifactorial nature of elite sport. While causal conclusions cannot be drawn due to the observational design, these results highlight the limitations of current anti-doping frameworks. They may still fall short in fully addressing performance enhancement, and ongoing refinement, considering physiological specificities is essential. Further research is warranted in this challenging field at the crossroads of human physiology, sports science, anti-doping, and sports regulation.

## Data Availability

The raw data supporting the conclusions of this article will be made available by the authors, without undue reservation.
