# Supplementary material for: The effect of doping, anti-doping measures, and sociological factors on the annual distribution of all-time top performances in short, middle, and long-distance running
Source: Front Sports Act Living. 2025 Nov 21;7:1683718. doi: 10.3389/fspor.2025.1683718 (PMC12678324; doi:10.3389/fspor.2025.1683718)
Supplement: Supplementary file 1 [file Datasheet1.docx]

Supplementary Material – Supplement

# Supplementary Data

One supplement included with this article, consisting of 2 tables and 1 figure.

# Supplementary Table S1

**Title: The total number of all-time top performances under the set time limit per category.**

Description: The table shows the total number of top performances per category included in the study, together with the time limits set for each category. This data also shows the total number of athletes who achieved top results in the selected period, as well as the percentage of top results achieved by the five athletes with the highest number of top results (top 5 athletes) in the defined subset in each discipline. The highest percentage of top results within the top 5 athletes is in the men’s and women’s 100m dash (almost 50%), while this percentage is around 30% in other categories.

Legend: 100m, 800m and 5000m – different running categories; number of top performances – total number of top performances under the set time limit; time limit – a time limit defined per category and per discipline; number of athletes – total number of different athletes achieving performance under the set time limit; percentage of top performances – the percentage of top performances achieved by the five athletes who have the highest number of performances under the set time limit; ratio of top performances – the ratio of the number of top performances achieved by the five athletes who have the highest number of top performances under the set time limit and total number of top performances under the set time limit

|  | | women | men |
| --- | --- | --- | --- |
| 100m | Number of top performances, (time limit) | 282, (<10.86 s) | 274, (<9.90 s) |
|  | Number of athletes | 50 | 52 |
|  | Top 5 athletes -  percentage of top performances, (ratio of top performances) | 47%, (133/282) | 49%, (134/274) |
| 800m | Number of top performances, (time limit) | 285, (<1:57.00 min) | 286, (<1:43.50 min) |
|  | Number of athletes | 84 | 79 |
|  | Top 5 athletes -  percentage of top performances, (ratio of top performances) | 32%, (92/285) | 28%, (96/286) |
| 5000m | Number of top performances, (time limit) | 257, (<14:38.00 min) | 250, (<12:57.00 min) |
|  | Number of athletes | 72 | 88 |
|  | Top 5 athletes -  percentage of top performances, (ratio of top performances) | 21%, (59/257) | 27%, (68/250) |

# Supplementary Table S2

**Title: Important parameters that lead to the choice of negative binomial regression as a regression model in this study.**

Description: The table presents alfa (α) as a dispersion parameter in negative binomial regression and its p value. In all categories α is different from zero which is confirmed with p value below 0.05, therefore negative binomial distribution has advantage over Poisson regression. Both zero-inflated negative binomial distribution (ZINB) and zero-inflated Poisson regression (ZIP) were tested. Zero-inflated Poisson regression was not shown to have an advantage over negative binomial regression according to the BIC value. Overall, zero-inflated negative binomial regression did not show significantly better delta BIC in comparison with negative binomial regression and in M100 and M5000 category the model was not concave pointing towards regular negative binomial regression as a better choice.

Legend: ^Δ^ – results from the year of inclusion in major official competitions to the end of data collection (1995-2023)
α - overdispersion parameter in negative binomial distribution
BIC - Bayesian information criterion, criterion for model selection
NB – negative binomial distribution, Poisson – Poisson's regression, ZIP – zero-inflated Poisson regression, ZINB – zero-inflated negative binomial distribution
n/a – parameter not applicable in specific category
W100m – women’s 100m discipline, W800m – women’s 800m discipline, W5000 – women’s 5000m discipline, M100m– men’s 100m discipline, M800m – men’s 800m discipline, M5000m – men’s 5000m discipline

| **category   model parameter** | **W100** | **W800** | **W5000^Δ^** | **M100** | **M800** | **M5000** |
| --- | --- | --- | --- | --- | --- | --- |
| **p value** | p=0.002 | p<0.001 | p=0.001 | p<0.001 | p<0.001 | p<0.001 |
| **α** | 0.154 | 0.245 | 0.134 | 0.385 | 0.389 | 0.424 |
| **BIC NB** | 225.665 | 274.746 | 193.501 | 212.661 | 277.047 | 210.365 |
| **BIC Poisson** | 232.392 | 291.000 | 200.624 | 243.199 | 308.805 | 246.476 |
| **BIC ZINB** | 224.132 | 265.295 | 192.994 | 214.668 | 259.167 | 207.097 |
| **BIC ZIP** | 229.956 | 280.506 | 198.027 | 240.283 | 268.604 | 273.989 |

# Supplementary Figure S1

Figure S1. The annual distribution of the results meeting the inclusion criteria annulled due to doping offense

The annual number of annulled results due to doping offenses, according to the alltime-athletics.com, is shown separately for women’s (a) and men’s (b) tested running categories. Since only few results per category met the inclusion criteria for the study all annulled results are shown. The exception was seen in women’s 100m running category where ten results met the inclusion criteria, and eight of them were achieved from year 2000 to 2003.

Legend:

W100m – women’s 100m discipline, W800m – women’s 800m discipline, W5000 – women’s 5000m discipline, M100m– men’s 100m discipline, M800m – men’s 800m discipline, M5000m – men’s 5000m discipline

N – the annual number results annulled due to the doping offense


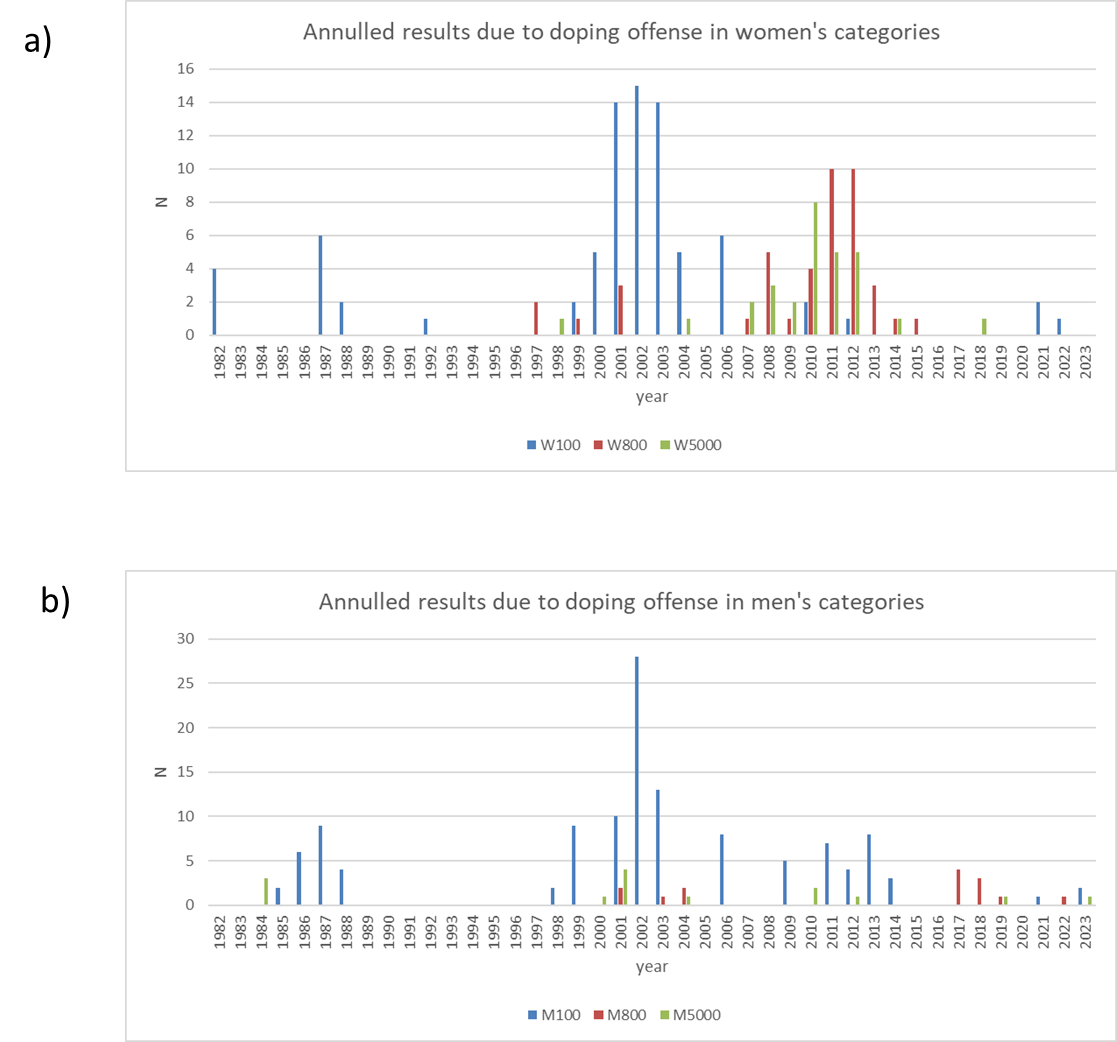


**
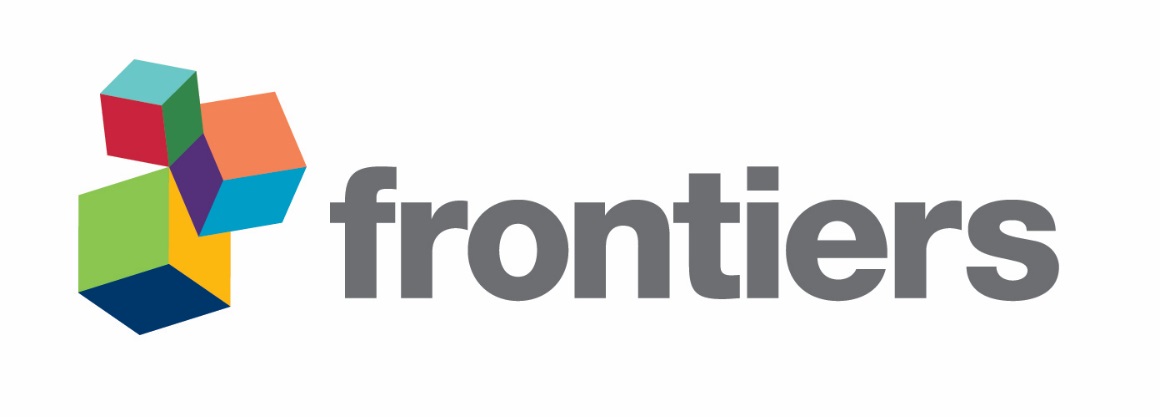
**
